# CD8 T Cell Vaccines and a Cytomegalovirus-Based Vector Approach

**DOI:** 10.3390/life11101097

**Published:** 2021-10-15

**Authors:** Marko Šustić, Maja Cokarić Brdovčak, Astrid Krmpotić, Stipan Jonjić

**Affiliations:** 1 Department of Histology and Embryology, Faculty of Medicine, University of Rijeka, 51000 Rijeka, Croatia; marko.sustic@uniri.hr (M.Š.); astrid.krmpotic@medri.uniri.hr (A.K.); 2 Center for Proteomics, Faculty of Medicine, University of Rijeka, 51000 Rijeka, Croatia; maja.cokaric@medri.uniri.hr

**Keywords:** CD8 T cells, T-Lymphocytes, vaccines, CMV, cytomegalovirus, vaccine vector

## Abstract

The twentieth century witnessed a huge expansion in the number of vaccines used with great success in combating diseases, especially the ones caused by viral and bacterial pathogens. Despite this, several major public health threats, such as HIV, tuberculosis, malaria, and cancer, still pose an enormous humanitarian and economic burden. As vaccines based on the induction of protective, neutralizing antibodies have not managed to effectively combat these diseases, in recent decades, the focus has increasingly shifted towards the cellular immune response. There is substantial evidence demonstrating CD8 T cells as key players in the protection not only against many viral and bacterial pathogens, but also in the fight against neoplastic cells. Here, we present arguments for CD8 T cells to be considered as promising candidates for vaccine targeting. We discuss the heterogeneity of CD8 T cell populations and their contribution in the protection of the host. We also outline several strategies of using a common human pathogen, cytomegalovirus, as a vaccine vector since accumulated data strongly suggest it represents a promising approach to the development of novel vaccines against both pathogens and tumors.

## 1. Introduction

The twentieth century was the golden age of vaccine development with the introduction of national vaccination programs on a massive scale [1]. This policy proved to be extremely effective as the number of new cases of polio, pertussis, measles, and other major infectious diseases plummeted after the introduction of preventive vaccination [2]. Today, the World Health Organization (WHO) estimates that preventive vaccination annually saves millions of lives [3,4]. Not only does vaccination save millions of people from premature death and unnecessary suffering, it is also hugely beneficial in economic terms. Studies have shown that the net benefit of vaccination in a developed country such as the Unites States can be up to $68 billion [5]. In low- and middle- income countries, where infectious diseases are still the leading cause of child morbidity and mortality [6], an investment of $34 billion in vaccination is estimated to result in savings of up to $586 billion in the direct cost of illness alone [7].

Although the benefit of vaccination has been known since the cowpox experiments of Edward Jenner [8], the biological mechanisms conferring an increased protection against infectious agents came to light only with the advent of modern immunology. Since then, it has become apparent that the majority of currently administered vaccines mediate their protection via the generation of protective, neutralizing antibodies [2,9]. Unfortunately, vaccines inducing a humoral immune response have not managed to solve the problem of numerous infectious diseases that still pose a major public health threat on a global scale, since the annual number of new cases for HIV, malaria, and tuberculosis are estimated to be 1.5 million, 230 million, and 10 million, respectively [10,11,12]. Influenza causes periodic annual epidemics with millions of cases. Moreover, Hanta viruses, Ebola, novel Flaviviruses such as Chikungunya and Zika, not to mention coronaviruses, such as Sars-CoV-2 which caused the COVID-19 pandemic, all present major threats to public health.

The bulk of effort in vaccine development has been received by humoral based immunization approaches [13]. However, as the intricacies of CD8 T cell biology became better known, a benefit of utilizing their cytotoxic properties and breadth of their epitope targeting for developing successful vaccines became apparent [14]. There is substantial evidence implicating CD8 T cells as the major determinant of protection not only against numerous viral and bacterial pathogens, but also in the fight against neoplastic cells [15,16,17]. Therefore, in this review we discuss the current evidence implicating CD8 T cells as important targets of vaccination strategies against pathogens and tumors. We also discuss the heterogeneity of memory CD8 T cell populations and their importance in the protection of the host. Finally, we describe several different attempts to use a common human pathogen, cytomegalovirus (CMV), as a vaccine vector with excellent properties for the induction of a potent and effective CD8 T cell response against vectored antigens. Although adequate CD8 T cell response cannot be generated without CD4 T cell help and this has to be taken into account when designing effective vaccine strategies [18], in this review, we focus exclusively on CD8 T cell biology.

## 2. T Cell vs. B Cell Vaccines

Do we really need CD8 T cell-based vaccines? To start answering this question, we first have to look at the evidence showing a memory CD8 T cell mediated control of infectious pathogens. Activated antigen specific CD8 T cells recognize microbial derived epitopes in complex with MHC-I molecules on the surface of infected cells. Upon recognition, CD8 T cells produce cytokines such as IFNγ and TNFα that induce widespread anti-microbial state, or, more locally, these CD8 T cells secrete cytotoxic molecules such as perforins and granzymes that initiate an apoptosis of the targeted cell [19,20]. Countless studies demonstrated CD8 T cell mediated pathogen clearance in animal models of infections. Memory CD8 T cells protect mice against several viral infections, including lymphocytic choriomeningitis virus (LCMV) [21], respiratory-syncytial virus (RSV) [22], and vaccinia virus (VV) [23]. In a mouse model of influenza infection, the loss of CD8 T cells resulted in a delayed viral clearance and host recovery [24,25]. Chronic viral infections with rapidly mutating viruses, such as HIV, pose a greater challenge for CD8 T cells. Despite that, rhesus macaques control simian immunodeficiency virus (SIV) spread via CD8 lymphocytes during acute infection, but also prevent SIV viremia surge during chronic infection as the depletion of this population has led to a rapid and substantial increase in viremia [26]. The importance of memory T cells was also implicated in parasitic infections. In a study by Schmidt et al., the authors induced large numbers of CD8 T cells specific for an immunodominant *Plasmodium berghei* epitope via heterologous prime-boost vaccination with antigen coated DCs and attenuated *Listeria monocytogenes* expressing the same epitope and managed to induce immunological protection against malaria challenge for at least 19 months [27].

Although most of the data accumulated so far on the relevance of the CD8 T cell response were obtained on animal models, research conducted on human immune response in recent decades essentially validated most of these observations. For instance, virus-specific memory CD8 T cells can be detected in humans even decades after acute viral infections or immunization with live attenuated vaccines [28,29]. Furthermore, a study by Akondy et al. tracked CD8 T cell response after vaccination with yellow fever virus (YFV) vaccine and showed that the memory pool of CD8 T cells is maintained by quiescent cells that divide only sporadically, with a doubling time of less than once a year. These CD8 T cells were identified even a decade after vaccination and possessed an epigenetic profile that indicated their potential for rapid response upon pathogen re-encounter [30]. The results obtained from several clinical studies of human influenza A (IAV) infection also corroborate the findings from the mouse model. An analysis of 342 adult individuals in a household control cohort study demonstrated an inverse correlation of pre-existing CD8 T cells directed against conserved influenza A epitopes and clinical symptoms associated with IAV infection [31]. Moreover, it has been shown that CD8 T cell numbers correlated with recovery from H7N9 influenza infection [32]. When it comes to HIV infection, the first indicators of CD8 T cell importance in suppressing viral replication came from GWAS studies. These studies identified *HLA-B* and *HLA-C* loci as almost entirely determining variations of HIV control in infected individuals [33]. A substantial enrichment in the frequency of HLA-B^*^5701 allele has been seen in elite HIV controllers compared to chronic progressors [34]. Beside genetic associations, studies examining cell functionality demonstrated that CD8 T cells isolated from controllers consistently showed a higher proliferative and cytolytic potential than cells isolated from patients with progressive infection [35,36]. Finally, in silico modeling of HIV mutational landscape showed that regions of structural Gag protein targeted by CD8 T cells of elite controllers have lower mutational tolerance than those targeted by progressors [37,38,39]. This implies that targeting conserved, essential sequences in HIV proteins is highly beneficial in protecting the host against the onset of AIDS. CD8 T cells are also strongly linked to protective immunity against *Listeria monocytogenes* [40], Ebola virus [41,42,43], and HCV [44,45], and to a lesser extent, against malaria [46], tuberculosis [47], and the currently raging Sars-CoV-2 virus [48].

Some peculiarities of CD8 T cell immunobiology, not present in humoral immune response, can be exploited to generate a vaccine against pathogens where B cell-based approaches have failed so far (Table 1). Neutralizing antibodies typically recognize heavily glycosylated structural proteins expressed on the outer surface of virions [23,49]. As these are highly variable proteins, humoral vaccines targeting them make the emergence of serological variants capable of escaping immune control very likely, especially in RNA viruses such as HIV or influenza. Conversely, in addition to structural proteins, CD8 T cells recognize epitopes derived from more conserved non-structural and internal proteins, making them better equipped to tackle mutated strains [50,51,52]. Moreover, CD8 T cells recognize a much broader repertoire of antigens than antibodies. In one of the earlier studies, an analysis of Immune Epitope Database and Analysis Resource (IEDB) revealed as much as 246 distinct epitopes restricted by MHC class I, and only 9 antibody epitopes in VV infection [23], with the majority of CD8 epitopes being derived from proteins expressed with early kinetics not found in the virions [53,54]. There is another intrinsic biological limitation imposed on humoral based vaccines, elegantly elaborated in recent publication by Stamper and Wilson [55]. Activation-induced cytidine deaminase (AID) generates somatic hypermutation during the germinal center reaction by deaminating cytosines. This enzyme does not target all cytosines equally, but rather has a propensity for deamination depending on the context within which these cytosines are located [56]. This means that immunoglobulin genes that have a higher percentage of deamination prone cytosines will mutate more frequently and have a higher likelihood of generating a favorable mutation which will be selected for during affinity maturation. As a result, the number of cytosines and the likelihood of favorable mutations will decrease as the germinal center reaction progresses. This has a very clear evolutionary benefit for the host as proven affinity-matured antibodies will be protected from detrimental mutations. However, it also limits the potential of memory B cells for re-diversification upon secondary antigen encounter. In this regard, further impediments for antibody diversification were identified in recent work by Mesin et al. [57]. They discovered that recall germinal centers are primarily composed of recruited naive clones and that memory B cells re-enter germinal center reaction only sporadically and hence rarely re-diversify. Altogether, these findings imply that antibody response upon antigen re-encounter becomes increasingly narrow and specialized, thus unable to cope with mutated variants. This is especially troublesome in infections with pathogens prone to mutations, such as HIV or influenza. On the other hand, memory CD8 T cell populations seem to have come up with a solution to tackle the evolutionary changes of pathogens. By enrolling cells with both high and low antigen affinities into memory CD8 T populations, high clonal diversity can be generated and maintained during extended periods of time, sufficiently broad to mount an effective and protective response against mutated pathogens [58,59,60]. Finally, it is worth stating that although intrinsic biological reasons exist for preferring one approach over the other, “T cell vs B cell” debate represents a case of false dichotomy as a perfect vaccine elicits both protective neutralizing antibodies and broad and effective T cell response.

## 3. Memory CD8 T Cell Subsets: The Division of Labor

**Figure 1:**
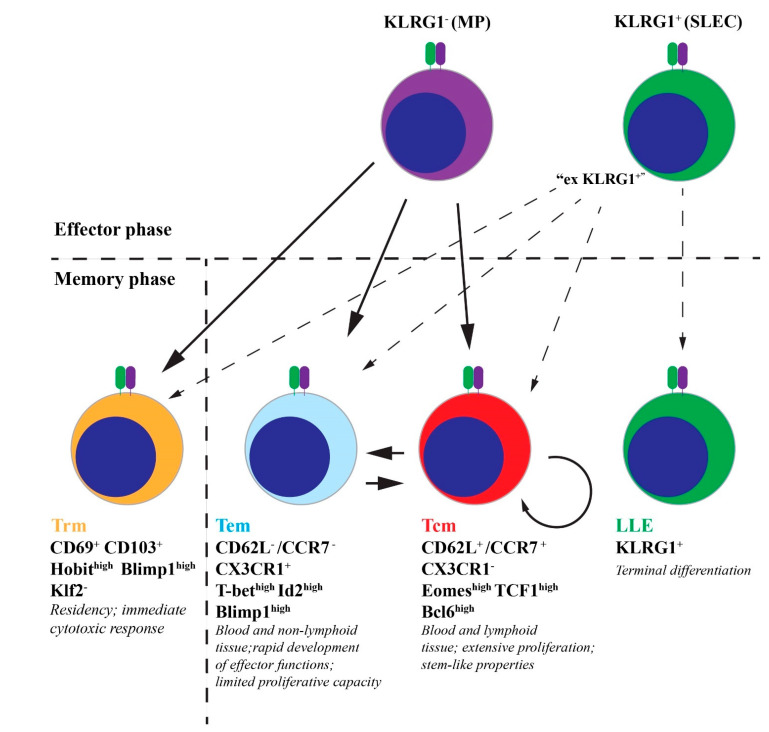
Differentiation and heterogeneity of memory CD8 T cells. Following the resolution of acute infection, the majority of effector CD8 T cells die off through the process of apoptosis. KLRG1^-^ cells preferentially survive and differentiate into all of memory CD8 T cell linages. However, some of the KLRG1^+^ cells lose the expression of KLRG1 (“ex-KLRG1^+^”) and also seed the memory compartment. A small proportion of terminally differentiated KLRG1^+^ cells survive as “long-lived effector” (LLE) cells. Memory compartment consists of resident memory (Trm), effector memory (Tem) and central memory (Tcm) cells. MP (memory precursor); SLEC (short-lived effector cells).

Long-term T cell-mediated protection depends upon the formation of a pool of memory cells ready to respond to future pathogen encounter. After pathogen clearance, the majority of effector CD8 T cells undergo apoptosis and only a small subset of memory cells persists long term in the mammalian organism (Figure 1). Natural selection has sculpted this population with a single goal in mind: to rapidly generate superior immunological responses upon re-encounter with pathogenic microorganism. What does this superior immunological response entail? Firstly, increased numbers of antigen specific cells capable of rapid and extensive proliferation upon antigen reencounter. Secondly, cells capable of immediate cytotoxicity and rapid cytokine production and finally, adequate anatomical distribution of effector cells in the tissue in which the pathogen re-encounter is most likely to occur. It seems that evolutionary processes have generated functional and anatomical heterogeneity in the CD8 T cell memory compartment roughly corresponding to these needs. The two traditional circulatory populations are central (Tcm) and effector memory (Tem) cells. Central memory cells express CD62L and CCR7 molecules, enabling their entry into secondary lymphoid organs, where the antigens draining from the tissue, if recognized as cognate, induce their rapid and extensive proliferation [61,62,63]. In contrast, initial studies classified Tem cells as CCR7^-^ CD62L^-^ and this pattern of chemokine and integrin receptor expression preferentially situates them in the bloodstream and non-lymphoid tissues. These cells show increased cytotoxicity and a rapid development of effector functions [61,64,65]. Transcription factors involved in the differentiation and maintenance of Tcm cells are Eomes [66], TCF1 [63], Id3 [67], Bcl6 [68], and Stat3 [69], while transcription factors linked to the formation of Tem cells include T-bet [70], Blimp1 [71], Id2 [72], and Stat4 [73]. More recent studies have identified several subpopulations within Tem compartment, including a KLRG1^+^, terminally differentiated population that likely originates from effector cells surviving the contraction phase of acute CD8 T cell response [64,74]. Further problems for this simple model of functional distinction emerged when Wherry et al. reported that there is no substantial difference in cytokine producing capabilities (with notable exception in IL-2 production) nor cytotoxicity between Tcm and Tem in acute LCMV infection [62]. On the other hand, several studies obtained different results where Tem cells showed higher cytotoxic capability and an increased protection against bacterial infection [75] and VV [76]. Interestingly, Tcm population was far superior against tumor challenge than Tem cells, due to its lymphoid tissue homing properties which entailed a rapid contact with the tumor antigen and subsequent proliferation and differentiation into effector cells [77]. The fact that different cellular populations confer an increased protection in different pathological conditions needs to be taken in consideration when constructing a CD8 T cell-based vaccine. It implies that “one size fits all” approach is most likely to fail and that CD8 vaccines need to be tailored to a specific pathogen or tumor. This is clearly demonstrated by the fact that in the study by Miller et al., the same population (KLRG1^+^ terminal-Tem) conferred excellent protection against *Listeria monocytogenes* but failed to even minimally affect the growth of murine melanoma [74].

The need for an adequate distribution of memory cells capable of immediate effector response in the peripheral tissue is supplied by tissue resident memory T cells (Trm). These cells remain locked in the tissue via the expression of CD69 and CD103 proteins. CD69 antagonizes the function of S1PR1 (sphingosine-1-phosphate receptor), thereby inhibiting the egress of resident cells from the tissue [78]. An important transcription factor for the expression of S1PR1 is KLF2 and this protein is inhibited in Trm cells by the Hobit transcription factor [79]. Other transcription factors that positively influence Trm formation are Runx3 [80], Notch [81], and Blimp1 [79,82]. Although it was initially thought that the defining characteristics of this population is the absence of migration, a recent study by Wijeyesinghe et al. revealed some degree of migration as these cells did seed the circulatory pool of Tem cells [83]. Regardless of their migration properties, Trm cells are poised for immediate effector functions producing high levels of Granzyme B and IFN-γ upon antigen encounter [84,85,86,87] and this population substantially contributed to the protection against a wide variety of infectious pathogens, such as herpes simplex virus [88], VV infection of the female reproductive tract [89], chlamydia [90], genital herpes [91], and others [92]. In a model of cutaneous skin infection with VV, the presence of Trm cells led to no less than a 10^4^ fold reduction in the viral load [93], demonstrating just how effective Trm cells can be in the protection of the host. Tissue resident memory cells were also shown to play an important role in the mouse melanoma model [94,95]. More importantly, a higher frequency of tissue resident cells was associated with increased survival in breast [96], lung [97], and ovarian cancers [98] (also reviewed in [99]). Several groups developed vaccination strategies that were able to induce large numbers of Trm cells poised to protect the tissue form infections and neoplastic growth. The immunization of mice via skin scarification with recombinant VV expressing OVA protein led to an enhanced protection against B16-OVA melanoma model [100] and against local vaccinia challenge [101]. Interestingly, both mucosal resident cells and circulating Tcm cells were needed to generate an adequate protective response against lethal respiratory VV challenge [101]. Tartour’s group developed a different approach, using non-replicative vaccine based on a B subunit of Shiga-toxin fused to tumor antigens. After mucosal immunization, but not intramuscular route, they induced CD49a^+^ and CD103^+^ Trm cells which inhibited the growth of orthotopic head and neck tumor and lung cancers [102,103]. Another approach to generate Trm cells was developed by Iwasaki’s group, called “prime and pull” method [91]. Instead of local antigen delivery, this method uses non-local immunization followed by local chemokine application to “pull” T cells to specific peripheral tissue. The authors immunized mice subcutaneously with attenuated HSV-2 strain and applied CXCL9 and CXCL10 chemokines topically to the vaginal cavity. T cells were recruited to the tissue with these chemokines via CXCR3 and established long-term residency which prevented the development of clinical diseases upon intravaginal challenge with a lethal HSV-2 virus. By comparison, only around 40% of animals immunized subcutaneously without the “pulling” of T cells to vaginal mucosa survived the challenge. The “prime and pull” strategy also led to substantially lower latent viral load in dorsal root ganglia, demonstrating the important role of resident cells in containing infection and preventing viral dissemination (also reviewed extensively in [104]).

## 4. Cytomegalovirus Based Vaccine Vectors

**Figure 2:**
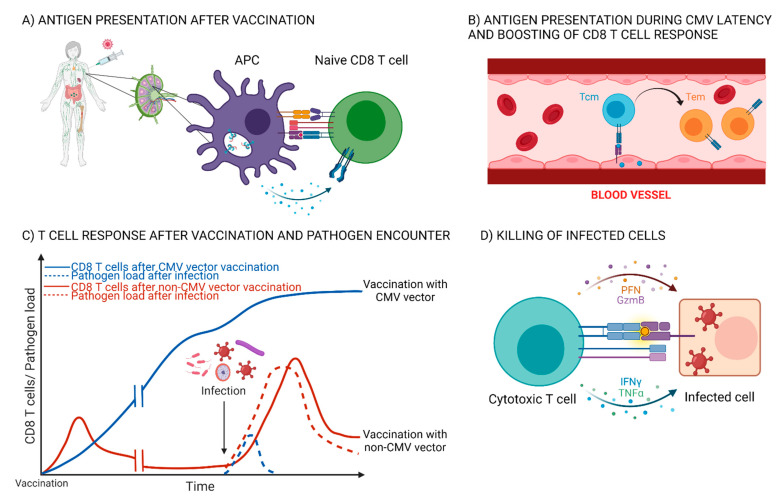
The model of CD8 T cell response induced with a CMV vector. (**A**) Following vaccination, dendritic cells in the draining lymph nodes present the vectored antigens to naïve CD8 T cells, which leads to proliferation and differentiation of CD8 T cells into multiple effector and memory lineages. (**B**) During CMV latency, sporadic viral reactivations boost CD8 T cell response, with accumulation of Tem cells, capable of immediate cytotoxic activity [105]. (**C**) CD8 T cell response upon immunization with a CMV vector compared to a vaccine that induces a “canonical” CD8 T cell response. CMV maintains high frequency of memory CD8 T cells specific for vectored antigens which allows for rapid pathogen control upon infection. (**D**) Upon recognition of pathogen derived antigens on the surface of infected cells, CD8 T cells secret perforins (PFN) which form pores in the cellular membrane of the targeted cell, allowing granzymes (Gzm) to enter the cytosol and initiate apoptosis. APC (antigen presenting cell); Tem (T effector memory); Tcm (T central memory). This figure was created with Biorender.

In the last two decades, it became evident that latently persistent viruses, such as CMV, generate a quite peculiar pattern of epitope specific T cell response, exemplified by the large numbers of cells exhibiting Tem phenotype with increased effector functions and without the signs of T cell exhaustion. These cells are maintained over the lifetime of the host, and importantly, the frequency of CD8 T cells specific for certain viral epitopes increases during the latency of the virus in a process known as memory inflation (reviewed in [105] see also [106,107]). This phenomenon was exploited by several groups in order to build a recombinant CMV based vaccine expressing exogenous antigens that would induce high numbers of effector cells, capable of a rapid control and elimination of infectious pathogens (Figure 2 and Table 2). Remarkable results were obtained by the groups of Luis Picker and Klaus Früh using this strategy in the protection against SIV, a virus closely related to HIV. They generated recombinant rhesus macaque CMV vectors (RhCMV) expressing several different SIV antigens and immunized rhesus macaques with a mixture of these vectors. In their initial study, the authors showed that RhCMV vectors expressing SIV antigens persistently infected monkeys, regardless of their pre-existing latent RhCMV infection. They were able to induce potent CD4 and CD8 T cell response, enriched in cells expressing Tem phenotype and animals vaccinated with these vectors showed increased resistance to repeated intrarectal SIV infection [108]. Next, they expanded on these findings and compared this approach to a vaccination protocol consisting of DNA vaccine and adenoviral vector that predominantly generates cells with Tcm phenotype. One year after vaccination, they intrarectally challenged macaques with a highly pathogenic SIV strain. Although the magnitude of CD8 T cells induced with different vaccination strategies were comparable at the end of vaccine phase, 13 out of 24 macaques immunized with RhCMV vectors rapidly controlled SIV spread while all of the DNA/adenovector vaccinated monkeys showed progressive infection. Protection correlated directly with Tem frequency, and interestingly, seemed to show an “all or nothing” pattern as macaques vaccinated with RhCMV vectors either completely controlled viral spread or exhibited the same level of viremia as unvaccinated controls [109]. It was later discovered that SIV is not immediately controlled at the entry site of vaccinated animals, but rather establishes infection in draining lymph nodes, bone-marrow, spleen, and liver. However, using an ultra-sensitive nested PCR 69–180 weeks after viral challenge, the authors failed to detect viral genome in these tissues in RhCMV vaccinated monkeys and when they adoptively transferred hematolymphoid cells from vaccinated animals to naïve hosts, no signs of infection were observed in the recipients [110]. Together, these results strongly indicated that despite breaking through the mucosal barrier and establishing productive infection in the tissues, SIV was controlled and completely cleared via Tem CD8 cells from the majority of vaccinated monkeys. In their subsequent studies [111,112], the authors analyzed epitope distribution and restriction patterns of CD8 T cells induced with RhCMV vectors, making several surprising discoveries. Firstly, CD8 T cells induced with RhCMV vectors had much broader epitope distribution than natural controllers or monkeys immunized with different vaccination modalities, recognizing on average 40 epitopes (average in other groups was 10–18). Secondly, all of the epitopes in RhCMV vacinated group where MHC-II or MHC-E restricted, without any canonical MHC-I epitopes. The loss of several genes in RhCMV viral strain used as a backbone for the vectors was responsible for this phenomenon and the restitution of these genes led to the loss of this heterodox restriction pattern [112]. Finally, they also discovered the crucial role of VL9 peptide embedded within Rh67 viral protein in MHC-E restricted CD8 T cell priming, as the loss of this gene resulted in failure of RhCMV vectors in protection against SIV challenge [113]. Hansen et al. also developed RhCMV vectors carrying *Mycobacterium tuberculosis* antigens. Immunization with these vectors resulted in significantly greater protection against pulmonary tuberculosis compared to unvaccinated animals, but also as compared to BCG immunized monkeys [114]. Finally, the same strategy was tried against malaria, but with modest success [115]. Although this strategy is a very promising approach to HIV vaccine development, this research might have a much broader and important implication for T cell-based vaccines. For some MHC-E epitopes, all of the vaccinated monkeys developed a CD8 T cell response, regardless of MHC-I haplotype. Due to the very limited MHC-E polymorphism in human population [116,117], it is quite plausible to presume that a vaccine targeting MHC-E restricted CD8 T cells would induce an immune response in all vaccinated individuals, thereby bypassing the perennial obstacle of MHC-I polymorphism in generating a successful, widely applicable CD8 T cell vaccine. After twenty years of development and preclinical studies, this approach is finally being tested in a Phase 1a clinical trial, in which CMV seropositive healthy individuals are being vaccinated with an HCMV expressing HIV antigens, which will be followed by Phase 2 and 3 trials in which the efficacy of this strategy will be determined [118].

A different strategy was investigated by the Cicin-Sain group. Instead of targeting Tem cells, the authors focused on the induction of protective Trm cells within the lungs. They generated an MCMV vector expressing an IAV epitope derived from hemagglutinin and intra-nasal immunization with this vector conferred substantial protection against IAV challenge [119]. A CMV vector was also utilized to develop a vaccination strategy against another major public health threat, Ebola. Ebola predominantly spreads to human populations from the infected carcasses of great apes, and Jarvis et al. argued that using a vector carrying Ebola antigens that can rapidly spread through targeted monkey populations can prevent Ebola outbreaks from skipping to humans. As a “proof of concept” they developed a mouse CMV vector carrying a CD8 T cell epitope from Ebola’s nucleoprotein (MCMV/ZEBOV-NP_CTL_) and this vector was able to confer excellent protection of mice against lethal Ebola challenge [120]. After this initial study, the authors also developed an RhCMV expressing Ebola virus glycoprotein and successfully vaccinated rhesus macaques with this vector, conferring to vaccinated animals substantially greater survival against lethal Ebola challenge compared to their unvaccinated counterparts [121]. Whether immunity following animal-to-animal spread will be sufficiently potent to protect the animals against Ebola induced disease still needs to be established. Finally, Nejad et al. constructed MCMV vectors expressing human papilloma virus (HPV) antigens, E6 and E7. By experimenting with different dosages and inoculation routes, they determined the threshold of tumor specific CD8 T cell response necessary for a complete protection against a tumor cell line harboring HPV antigens. Importantly, the authors demonstrated that high levels of pre-existing immunity to MCMV hamper subsequent immune response to an MCMV vector carrying tumor epitopes which leads to the loss of protection against subcutaneous tumor challenge [122]. Additionally, CMV vectors targeting melanoma antigens such as gp100 and TRP2 [123,124] and human prostate specific antigens (PSA) [125] were also developed.

In vaccine development, considerations of safety are as important as those of immunogenicity. Our group developed an attenuated but highly immunogenic vaccine vector based on mouse cytomegalovirus equipped with a ligand for an activating NKG2D receptor. The rationale behind this approach was the activating role of NKG2D on NK cells, which would provide a quick control of a vector carrying an NKG2D ligand, but also a costimulatory role of this receptor on CD8 T cells [126]. We inserted an NKG2D ligand RAE-1γ in place of its viral inhibitor m152 (RAE-1γMCMV) and this virus proved to be highly attenuated compared to wild-type virus, but despite this, induced an equal CD8 T cell response to inflationary m164 and pp89 epitopes and comparable amounts of circulating antibodies against MCMV. Neutralizing antibodies generated with RAE-1γMCMV were transferred through the placenta to fetuses of pregnant female mice and were able to protect newborns against pulmonary and brain MCMV infection. RAE-1γMCMV virus was attenuated in an NKG2D dependent manner, as blockade of NKG2D using monoclonal antibodies led to a loss of attenuation. Additionally, this virus remains stable in the host over extended periods of time without the loss of the RAE-1γ gene [127]. In our further research, we inserted a foreign epitope, *Listeria monocytogenes* derived LLO epitope or ovalbumin derived SIINFEKL, into the RAE-1γMCMV backbone. Irrespective of the attenuation, RAE-1γMCMV generated a superior CD8 T cell response to inserted foreign epitopes and conferred a superior protection against *Listeria monocytogenes* infection. Surprisingly, this phenomenon was not lost in *Klrk1^-/-^* animals, suggesting that either RAE-1γ has an unknown interaction partner or that superior CD8 response is independent of ectopic RAE-1γ expression [128]. We also tested the protective capacity of RAE-1γ expressing vector against subcutaneous tumor challenge and demonstrated its excellent anti-tumor capabilities [129]. Recently, we performed a detailed characterization of memory CD8 T cells induced with RAE-1γMCMV vector expressing the SIINFEKL epitope. A transcriptomic analysis of RAE-1γMCMV primed CD8 T cells showed a terminally differentiated, effector like phenotype with higher expression of effector genes such as *Cx3Cr1* and *Gzm B*, while wild-type (MCMV-SIINFEKL) primed cells had a higher expression of prototypical Tcm genes, *Sell* and *Ccr7*. Interestingly, cells positive for TCF1 transcription factor (crucially associated with Tcm phenotype, stemness and long-term persistence [130]) were more numerous in MCMV-SIINFEKL primed CD8 T cells, but the difference was observed only at memory time-points, beginning around day 30. These phenotypical differences also entailed functional distinctions, but upon adoptive transfer of equal numbers of CD8 T cells into mice harboring subcutaneous tumors, cells primed with both vectors showed similar tumor-rejecting capabilities [131]. Finally, we developed an MCMV viral vector containing another NKG2D ligand, MULT-1. MULT-1 has the highest affinity for NKG2D and demonstrated even higher attenuation than RAE-1γMCMV. Cellular immune response for inserted foreign epitope was somewhat inferior to RAE-1γ expressing vector, but the humoral response was comparable and provided equal protection to newborns of vaccinated mothers against congenital MCMV infection [132]. The translational potential of CMV vector expressing an NKG2D ligand was confirmed by generating a human CMV (HCMV) armed with a human homologue of RAE-1γ, ULBP2 [133]. Here, the TB40 strain which lacks several immunoevasive genes such as US2, US3, and US6 whose function is to interfere with MHC-I expression on the surface of infected cells, was used as a vector backbone. ULBP2-HCMV was attenuated via NK cells and NKG2D receptor but showed comparable humoral and T cell response to viral antigens in humanized mice models. These results demonstrate the feasibility of HCMV based vector vaccines, but several points need to be highlighted. Since HCMV shows high prevalence in the human population, a necessary precondition for a wide use of CMV based vectors is a capability of these vectors to superinfect HCMV positive hosts. Hansen et al. showed that precisely the RhCMV homologs of US2, US3 and US6 are necessary for superinfection of RhCMV positive rhesus macaques [134]. If these results obtained from non-human primates can be extrapolated to humans, the deficiency of viral immunoevasins in ULBP2-HCMV needs to be further addressed.

## 5. Conclusions

The heightened interest in generating CD8 T cell-based vaccines in recent years has coincided with an expansion of our understanding of basic molecular features in CD8 T cell immunobiology. The advances of single cell-sequencing technology and mass-cytometry have revealed substantially greater heterogeneity in the CD8 memory compartment than previously thought and using these novel technologies in identifying a population best suited to tackle a given pathological condition can have immense impact on vaccinology. Complementing this with an increased understanding of host–virus interactions and the modulation of immune response by viral genes presents us with a promising approach for using recombinant viral vectors, genetically manipulated to change the pattern of the CD8 T cell response. In this regard, CMV presents an ideal viral vector candidate as its lifelong persistence maintains a high number of functional effector CD8 T cells against vectored antigens without the need for booster doses. We believe that utilizing this virus as a vector can easily find its way to clinical applications against both microbial and neoplastic threats.

## Figures and Tables

**Table 1:** Differences in antigen targeting between CD8 T cells and B cells.

	CD8 T Cells	B Cells
**Targeted antigens**	Structural and non-structural proteins [50,51,52]	Surface proteins [23,49]
**Spectrum of antigens**	Broad [23,53,54]	Narrow [23]
**Response upon antigen** **re-encounter**	High clonal diversity [58,59,60]	Focused and specialized [57]

**Table 2:** Cytomegalovirus (CMV)-based vaccine vectors.

Vaccine Vector	Inserted Antigen	Disease (Challenge) Model	Immune Response	Selected References
RhCMV/SIV	Env, gag, rev-tat-nef (fusion protein)	Highly pathogenic SIV strain intra-rectal challenge	CD8 T_EM_ cell response	[108,109,110,111,112,113]
RhCMV/TB	Ag85A, Ag85B, Rv3407, Rv1733, Rv262, Rpf A, Rpf C, Rpf D, ESAT-6	*Mycobacterium tuberculosis* Erdman strain intrabronchial challenge		[114]
RhCMV/Pk	CSP, AMA1, SSP2/TRAP, MSP1c	*Plasmodium knowlesi* sporozoites challenge		[115]
MCMV/ZEBOV-NP_CTL_	CD8 T cell epitope from the nucleoprotein (NP) of *Zaire ebolavirus*	Mouse adapted ZEBOV challenge		[120]
MCMV^IVL^	MHC-I restricted IVL_533–541_ epitope	Lethal IAV PRM8 variant challenge	CD8 T_RM_ cell response	[119]
MCMV/PSA_FL_	full length human prostate-specific antigen ORF	Prostate cancer model (TRAMP-PSA tumor cells)	PSA_65–73_ inflationary CD8 T cell response	[125]
MCMV/PSA_65–73_	MHC-I restricted PSA_65–73_ epitope			
RAE-1γMCMVList	MHC-I restricted epitope of *Lysteria monocytogenes* listeriolysin O_91–99_ (LLO)	Hemolytic EGD strain (serovar1/2a) of *Lysteria monocytogenes* challenge	Protective CD8 T cells	[128]
RAE-1γMCMV-SIINFEKL	MHC-I restricted peptide SIINFEKL	B16OVA (melanoma) and EG7 (thymoma) model		[129,131]
MCMV-M79-FKBP-E7	HPV16 E7_49–57_ epitope	Subcutaneous administration of TC-1 cells transformed with HPV16 E6/E7 and c-H-ras oncogenes	Tumor-specific protective CD8 T cells	[122]
MCMV-gp100^S27P^	gp100_25–33_ peptide (gp100^S27P^)	Subcutaneous B16F0 melanoma model	Tumor-infiltrating antigen-specific CD8 T cells	[123]
RhCMV/EBOV-GP	Codon-optimized full-length EBOV GP	Lethal NHP EBOV challenge	IgG responses correlated to protection, but with no neutralization capacity	[121]
MCMV-TRP2	Mouse tyrosinase-related protein 2 melanoma antigen	B16 melanoma challenge	IgG antibody-mediated tumor protection	[124]
